# The effectiveness of text message-based self-management interventions for poorly-controlled diabetes: A systematic review

**DOI:** 10.1177/2055207617740315

**Published:** 2017-11-09

**Authors:** Rosie Dobson, Robyn Whittaker, Leila Pfaeffli Dale, Ralph Maddison

**Affiliations:** 1 National Institute for Health Innovation, University of Auckland, New Zealand; 2 Institute for Innovation and Improvement, Waitemata District Health Board, New Zealand; 3 School of Kinesiology, University of British Columbia, Canada; 4 Institute for Physical Activity and Nutrition, Deakin University, Australia

**Keywords:** mHealth, diabetes mellitus, mobile phone, text message, review

## Abstract

**Background:**

Poorly controlled diabetes leads to debilitating complications at a significant cost to health systems. Text messaging is an ideal platform for the delivery of self-management interventions to patients with poorly controlled diabetes due to the ubiquity of mobile phones, and the ability of text messaging to reach people in their everyday lives when self-management of the condition is vital. This systematic review aimed to assess the effectiveness of short message service-based diabetes self-management interventions on glycaemic control in adults with poorly controlled diabetes.

**Methods/design:**

MEDLINE, PubMed, EMBASE, The Cochrane Library and PsychINFO were searched from inception through to 23 January 2017 for randomised controlled trials investigating the use of text messaging based self-management interventions on haemoglobin A1c for patients with poorly controlled diabetes.

**Results:**

Seven studies met the inclusion criteria and were included in the review. Three of the studies reported a significant decrease in haemoglobin A1c from baseline to follow-up in the intervention group compared to the control group. No clear relationship between positive outcomes and intervention dose, content and functionality was seen.

**Discussion:**

Evidence supporting text messaging for improvements in glycaemic control in people with poorly controlled diabetes is mixed. Previous reviews have reported positive impacts on glycaemic control for short message service interventions in patients with diabetes; however, when limited to those with poorly controlled diabetes the evidence is less clear. Large-scale studies with robust methodology and longer-term follow-up are needed to further understand the impact of text-messaging-based self-management interventions for people with poorly controlled diabetes.

## Introduction

Addressing the growing global burden of diabetes is a priority for health services. There is considerable evidence that good glycaemic control in patients with both type 1 or type 2 diabetes results in significant reductions in the risk of developing complications, such as renal failure, diabetic retinopathy, lower limb amputation, stroke and heart disease.^1–7^ These complications not only have an detrimental impact on a patient's quality of life, but also the clinical management of these is a significant source of health service expenditure.^8^ A haemoglobin A1c (HbA1c) target of < 7% (<53 mmol/mol) is the widely recommended target for good control.^9,10^ When glycaemic control is sub-optimal (>7%; 53 mmol/mol) or poor (>8%; 64 mmol/mol) increased intervention is recommended.^11^ Estimates indicate that approximately 25–30% of people with diabetes have HbA1c levels over 8% (64 mmol/mol) indicating poor control, and higher rates are seen in ethnic minorities.^12,13^ Given the costly and debilitating nature of both the microvascular and macrovascular complications of poorly controlled diabetes, considerable support and input is needed to achieve and maintain this target of good glycaemic control.

Individual behaviours play an integral role in diabetes control including blood glucose monitoring, medication adherence, healthy eating and physical activity, and therefore diabetes self-management education and support is a fundamental part of diabetes care. There is a wide range of interventions designed to support people to self-manage their diabetes; from passive interventions (e.g. provision of information) to more active interventions (e.g. interventions to change behaviour or increase self-efficacy).^14^ Supporting a person's self-management of their condition may involve providing encouragement and information to help that person obtain greater control of their condition. Support may increase a person's understanding of their condition, encouraging them to be active participants in the decision making around their condition and motivating them to engage in healthy behaviours.^14^ Interventions designed to support diabetes management have traditionally been delivered via written materials or in face to face or group sessions such as Diabetes Self-Management Education (DSME) programmes. DSME is designed to address the seven key self-management behaviours identified by the Association of American Diabetes Education; (a) healthy eating, (b) being active, (c) monitoring, (d) taking medication, (e) problem solving, (f) reducing risks, and (g) healthy coping.^15^ For patients with poor control, however, support may need to extend beyond traditional healthcare settings to sustain the behaviours needed to manage diabetes in the context of a patient's daily life. There is growing evidence for the use of mobile phones for this purpose.

Use of the short message service (SMS), or text messages, has the advantage of instant transmission at a low cost to end users and, given the ubiquity of mobile phones, could be an ideal platform for the delivery of diabetes self-management support. Previous systematic reviews have provided support for the effectiveness of mobile health (mHealth) for diabetes self-management,^16–18^ although these reviews have included studies of patients without specifying a level of glycaemic control (i.e. including those who are already maintaining good control of their diabetes). It is our understanding that no previous review has specifically looked at the use of SMS in patients with the greatest need (i.e. not meeting the recommended HbA1c target). The purpose of this systematic review was to evaluate the current evidence for the use of SMS to deliver diabetes self-management interventions to improve glycaemic control in adults with poorly controlled diabetes. Specific aims included; (a) to examine the effectiveness of SMS-based diabetes self-management interventions on change in HbA1c, (b) to explore the theoretical basis of these interventions and commonly utilised behaviour change techniques (BCTs),^19^ and (c) to understand the features/components of these SMS interventions that are associated with better outcomes in this population.

## Methods

This systematic review was conducted according to the Preferred Reporting Items for Systematic Reviews and Meta-Analyses (PRISMA) checklist^20^ (see Supplementary Material, Appendix 1 for the completed checklist). The protocol was not published.

### Eligibility criteria

Eligible studies were randomised controlled trials (RCTs) utilising SMS messages to deliver diabetes self-management interventions to adults with poorly controlled diabetes. Participants of eligible studies were adults aged 16 years and over with poorly controlled diabetes (type 1 or 2), defined as HbA1c over 7% (53 mmol/mol). Although the definition of poor control is generally considered to be >8% (64 mmol/mol), few studies have specifically targeted this group. Therefore, for the purpose of this review, it was decided that studies targeting only those patients not meeting the widely accepted standard for good diabetes control be included.^9,10^ Studies that examined mixed chronic disease populations or pregnant patients were excluded.

Studies in which SMS was the platform for delivering diabetes self-management interventions (education, reminders, monitoring, self-care i.e. nutrition, exercise) were included. Studies with multifaceted interventions where SMS was just one component of the intervention were included in the review if SMS was a primary component that all intervention participants received. Studies were excluded if they examined the use of messages created by a clinician/investigator based on individual clinical judgement or where SMS was used only as a means of real-time communication between provider and patient (i.e. not an automated programme). Studies were included if the comparator or control group involved either no intervention (usual care) or an intervention variant that did not include SMS. Included studies needed to report HbA1c as a measure of diabetic control as a primary or secondary outcome.

The review was restricted to full-text articles published in peer reviewed journals. Studies were excluded if published in languages other than English or were published only in the form of conference abstracts.

### Search strategy

Comprehensive searches were conducted from inception through to 23 January 2017 using MEDLINE, PubMED, EMBASE, The Cochrane Library and PsychINFO. Details of the MEDLINE search strategy can be seen in Table 1 (amended for other databases). Reference lists of relevant previous reviews and included studies were searched for additional papers.

**Table 1.** MEDLINE search strategy.

#	Search	Results
1.	mobile phone/	7613
2.	(((mobile or smart) and phone*) or smartphone*).tw.	10,704
3.	(cell* and (phone* or telephone*)).tw.	4543
4.	(mhealth or m-health or mobile-health).tw.	2297
5.	((text or sms or short or instant) and messag*).tw.	5906
6.	(texting or texted).tw.	612
7.	1 or 2 or 3 or 4 or 5 or 6	22,042
8.	diabetes mellitus/	114,517
9.	diabet*.tw.	565,564
10.	(IDDM or NIDDM or MODY or T1DM or T2DM or T1D or T2D).tw.	41,514
11.	8 or 9 or 10	591,081
12.	7 and 11	1007

### Selection of studies

The searches were carried out by the first author and results merged into EndNote X7 Referencing Software where duplicates were removed. Titles and abstracts were screened and unrelated articles excluded. Articles identified for full text review were reviewed against the criteria above by the first author and any uncertainty around inclusion was resolved by consensus with the other authors. Reasons for exclusion were recorded.

### Data extraction

Data were extracted using structured forms informed by the PRISMA checklist^20^ and Cochrane Systematic Review Handbook,^21^ including; study design (design, duration), population characteristics (sample size, diabetes type, age, country), intervention (description, tailoring), comparator (description), theoretical model and outcomes. In addition, each study was assessed for use of BCTs and the diabetes self-management behaviours targeted. BCTs were coded using the BCT taxonomy (v1) of 93 hierarchically clustered techniques.^19^ During data extraction, we also evaluated whether the authors reported on an adequate randomization process, allocation concealment, whether outcomes assessors were blinded, attrition rate and whether there was evidence of selective reporting. Data extraction was performed by the first author and any uncertainty resolved by consensus following independent assessment by other authors. A narrative synthesis methodology was used to synthesise the data extracted.

### Risk of bias assessment

Risk of bias was assessed using methods outlined in the Cochrane Handbook for Systematic Reviews of Interventions for assessing the risk of bias^21^ for the following domains: selection bias (including method of randomization and allocation concealment), detection bias, attrition bias and reporting bias. If available, published study protocols and trial registry data were accessed to inform risk of bias assessment. Trial registry sites were searched if trial registration was not stated in manuscript. Risk was judged as high, low, or unclear. Unclear risk was given if there was a lack of information or uncertainty.

## Results

**Figure 1.**
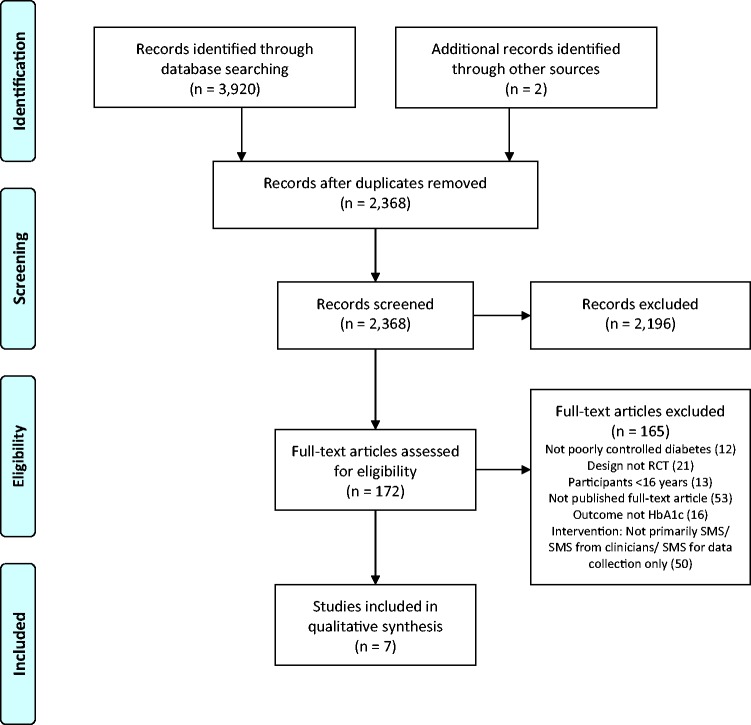
Preferred Reporting Items for Systematic Reviews and Meta-Analyses (PRISMA) flow diagram of study selection. RCT: randomised controlled trial.

A total of 3922 records were identified from the combined database searches and other sources. Once duplicates were removed, 2368 records were screened for eligibility using title and abstract. One hundred and seventy-two full-text articles were assessed for eligibility, of which seven studies met the inclusion criteria and are included in this review. Figure 1 shows the data collection process.

### Assessment of risk of bias

**Figure 2.**
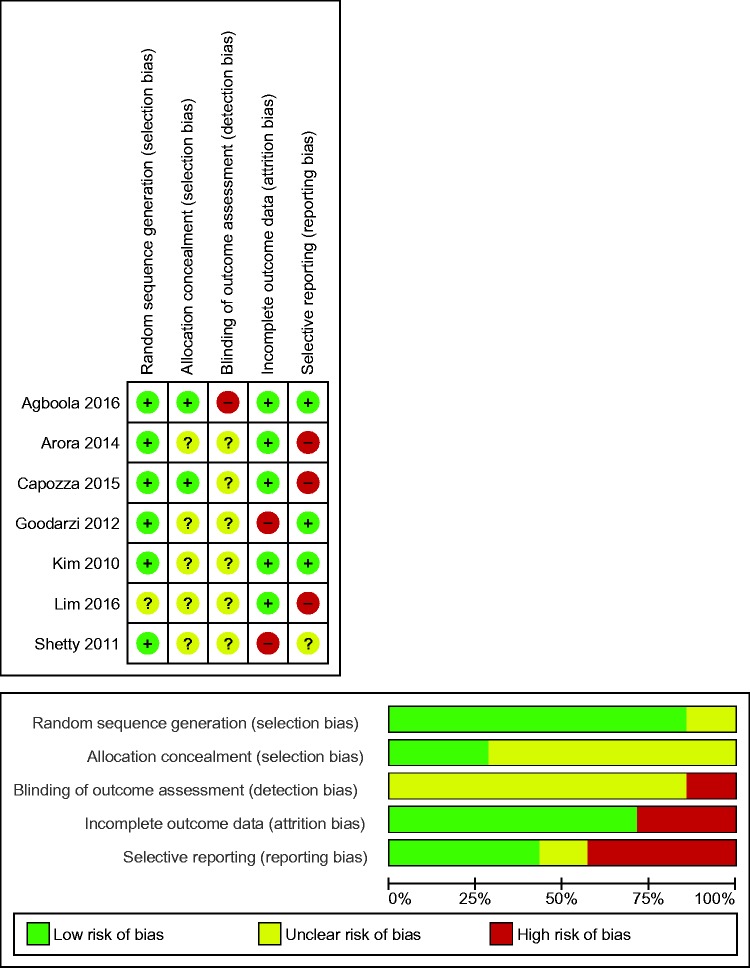
Risk of bias summary and graph: review authors' judgements about each risk of bias item for each included study and presented as percentages across all included studies.

Figure 2 presents the risk of bias summary and graph (see Supplementary Material, Appendix 2 for further detail of the judgements for risk of bias in the included studies). Inadequate reporting meant that presence of bias was unclear in all but one of the studies and therefore it could not be judged that any study was free of bias. Two studies were low risk for selection bias (low risk for sequence allocation and allocation concealment).^22,23^ Due to the nature of mHealth interventions, meaning participant blinding is not feasible, detection bias was determined on blinding of the outcome assessors only. Blinding was not described in six of the studies, with one study considered as high risk due to the absence of blinding.^22^ Two studies were considered as high risk for attrition bias with the remaining five studies considered as low risk.^22–26^ No study referenced a published protocol but all were registered with a clinical trials registry with the exception of one.^27^ For those registered, three studies were considered as low risk for reporting bias.^22,25,28^

### Characteristics of studies

The characteristics of the included studies can be seen in Table 2.^22–28^

**Table 2.** Characteristics of included studies.

Study	Study design	Participants: Sample size (baseline/follow up); age (mean (SD)); country	Intervention		Comparison group	Theoretical model
			Description	Tailoring		
Agboola (2016)^22^	RCT Parallel 2 arms 6 months	126/95 adults with T2D (HbA1c>7%) Age: IG=50.3 years (10.5); CG=52.6 years (12.6) Country: USA	‘Text to Move (TTM)’ – SMS and pedometer. Two SMS per day at set times; one providing coaching/feedback based on daily step counts received via pedometer and pre-set goals; and the other support, health education, motivation, and reminders to engage in healthy behaviours. Data stored on a web-based portal viewable by participants. Versions in English and Spanish.	Stage of behaviour change and language	Usual care and pedometer	Transtheoretical model
Arora (2014)^24^	RCT Parallel 2 arms 6 months	128/92 adults with T2D (HbA1c≥8%) Age: IG=50.5 years (10.3); CG=51.0 years (10.2) Country: USA	‘TExT-MED’ – two SMS per day at set times providing education/ motivation, medication reminders, healthy living challenges and diabetes trivia. Versions in English and Spanish.	Language	Usual care	Health belief model
Capozza (2015)^23^	RCT Parallel 2 arms 6 months	156/93 adults with T2D (HbA1c>8%) Age: IC=52.0 years (11.2); CG=54.5 years (10.7) Country: USA	‘Care4Life’ – Bidirectional SMS with web-based portal for viewing response trends from reply SMS. Between 1–7 SMS per day including one diabetes education SMS each day. Optional SMS including medication reminders, glucose testing reminders, BP monitoring reminders, and tracking and encouragement toward weight loss and exercise goals. Versions in English or Spanish.	Message types, frequency and language	Usual care	None reported
Goodarzi (2012)^28^	RCT Parallel 2 arms 3 months	100/81 adults with T2D (HbA1c>7%) Age: IG=51.0 years (10.3); CG=57.0 years (9.8) Country: Iran	Four SMS per week with information about exercise, diet, diabetic medication, and self-monitoring of blood glucose.	None reported	Usual care	None reported
Kim (2010)^25^	RCT Parallel 2 arms 3 months	100/92 adults with T2D (HbA1c 7-12%) Age: IG=47.8 years (9.6); CG=49.0 years (10.7) Country: Korea	Web based system which receives data from glucometer and sends automatic adjustments of insulin dose via unidirectional SMS each day at 17:00.	Insulin adjustments based on patient-specific data	Usual care and glucometer	None reported
Lim (2016)^26^	RCT Parallel 2 arms 6 months	100/85 adults with T2D (HbA1c 7-10.5%) Age: IG=64.3 years (5.2) CG=65.8 years (4.7) Country: Korea	CDSS-based U-Health Service which receives data from activity monitor and glucometer, and then CDSS rule engine sends SMS feedback and instructions including lifestyle changes and medication adjustments. In addition a website for recording diet and receiving dietary feedback.	Messages based on patient-specific data	Usual care, glucometer and pedometer	None reported
Shetty (2011)^27^	RCT Parallel 2 arms 12months	225/144 adults with T2D (HbA1c 7-10%) Age: IG=50.1 years (9.9) CG=50.5 years (8.3) Country: India	2-4 SMS per three days providing instructions on nutrition, physical activity, healthy living and reminders to follow medication prescription.	Content and frequency	Usual care	None reported

BP: blood pressure; CDSS: clinical decision support system; CG: control group; HbA1c: haemoglobin A1c; IG: intervention group; RCT: randomised controlled trial; SD: standard deviation; SMS: short message service.

#### Study design and participants

All of the included studies were two-arm parallel group randomised controlled trials. The study durations were three months,^25,28^ six months,^22–24,26^ or 12 months.^27^ In four studies the comparator was usual care alone,^23,24,27,28^ and in the remaining studies there was usual care with the addition of either a glucometer,^25^ pedometer,^22^ or both.^26^ Three of the studies took place in the USA,^22–24^ two in Korea,^25,26^ one in India^27^ and one in Iran.^28^

All of the studies included adults with type 2 diabetes. In the majority of the studies participants were required to have a baseline HbA1c level over 7%,^22,25–28^ with only two stating that they were targeting those with poorly controlled diabetes requiring HbA1c over 8% (64 mmol/mol).^23,24^ Baseline sample sizes ranged from 100–225 and included a total of 935 participants. Participants were relatively homogenous in terms of mean age (late-40s to mid-50s) with the exception of one study in which the target population was older adults aged 60 years and over.^26^ High attrition was seen in four of the seven studies.^22–24,27^

#### Intervention

Only three of the studies utilised SMS as the sole intervention,^24,27,28^ the remaining studies included pedometers/activity monitors,^22,26^ glucometers,^25,26^ web-based tools^22,23,25,26^ and home gateway systems.^26^ All but one of the interventions were tailored to the participant to some degree. In two studies the message content and frequency was tailored by individual preferences,^23,27^ three allowed participants to choose the language of the messages,^22–24^ and three provided feedback on patient-specific data received from devices (glucometer or pedometer).^22,25,26^ One study tailored the content based on the participant's stage of change (transtheoretical model of behaviour change).^22^

SMS functionality varied in the studies. In two studies, SMS was used for providing education/information only,^27,28^ and in another two studies only feedback and treatment instructions were provided.^25,26^ In one study, SMS functioned as a tool to provide feedback, motivation and education,^22^ in another the SMS delivered education and reminders,^24^ and in the final study SMS functionality included education, reminders, data collection and feedback.^23^

The dose of SMS in the studies varied with two delivering less than one SMS per day,^27,28^ one study delivering one SMS per day,^25^ and two studies delivering two SMS per day.^22,24^ The dose was variable in two studies. In one, SMS were sent in response to incoming data which was requested a minimum of eight times per week,^26^ and in the other study participants selected the dose and could receive between 1–7 messages per day.^23^

There was considerable variation in the content of the SMS messages. Table 3 shows the frequency of studies addressing specific self-management behaviours identified by the Association of American Diabetes Educators.^15^ Two studies targeted single behaviours – physical activity^22^ or medication adherence.^25^ One study targeted three behaviours, two studies targeted four behaviours and two studies targeted five behaviours (see Table 3).

**Table 3.** Diabetes self-management behaviours targeted by interventions.

Self-management behaviours	Number of studies targeting behaviour
Healthy eating	5^23,24,26–28^
Physical activity	6^22–24,26–28^
Blood glucose monitoring	5^23,24,26,28^
Taking medication	6^23–28^
Problem solving	0
Reducing risks	1^24^
Healthy coping	1^23^

Self-management behaviours identified by the Association of American Diabetes Education.^15^

Only two of the seven studies explicitly stated that they had a theoretical basis: the transtheoretical model^22^ and health belief model.^24^ The most commonly utilised BCTs in the interventions were ‘4.1. Instruction on how to perform the behaviour’ and ‘5.1. Information about health consequences’. Other commonly utilised techniques included ‘2.4. Self-monitoring of outcome(s) of behaviour’, ‘2.7. Feedback on outcome(s) of behaviour’, and ‘7.1 Prompts/cues’. A summary of the frequency of BCTs utilised in the interventions can be seen in Table 4. In two of the studies, the control group were asked to perform self-monitoring of the outcome(s) of behaviour (BCT 2.4) but were not provided any feedback on this. The control arms of all other studies did not incorporate BCTs.

**Table 4.** Behaviour change techniques utilised.

Behaviour change technique^19^	Number of studies incorporating technique in the intervention
1.1 Goal setting (behaviour)	1^23^
1.5 Review behavioural goal(s)	1^22^
2.4. Self-monitoring of outcome(s) of behaviour	4^22,23,25,26^
2.7. Feedback on outcome(s) of behaviour	4^22,23,25,26^
4.1. Instruction on how to perform the behaviour	5^24–28^
5.1. Information about health consequences	5^22–24,27,28^
7.1 Prompts/cues	4^22–24,26^
8.1 Behavioural practice/rehearsal	3^22–24^

#### Outcomes

A significant decrease in HbA1c from baseline to follow-up in the intervention group compared to the control group was seen in only three of the seven studies.^25,26,28^ The remaining studies all showed a decrease in mean HbA1c in the intervention group from baseline to follow-up and this difference was significant in one study but not when compared to the control group.^22^

A summary of the key findings of the included studies can be seen in Table 5. There was very little consistency in other outcome measures reported in the studies. Four studies^22,24,26,27^ reported on changes in physical activity with only one study reporting a significant increase in the frequency of activity in the intervention group.^26^ Three studies reported on changes to diet, with one study reporting no significant changes in adherence to diet prescription,^27^ one study reported no significant change in diet behaviours,^24^ and the other showed a significant decrease in the mean caloric intake of the intervention group.^26^ Two studies^24,28^ reported on changes in diabetes-related self-efficacy, with both showing improvements in the intervention group but only one reporting a significant change in this construct in the intervention group compared with the control group.^28^ Diabetes knowledge was also reported in these two studies, again both studies showed improvements in the intervention group but only one reported a significant change compared with the control group.^28^ Two studies reported improvements in blood glucose monitoring in the intervention group,^24,25^ but in only one of these studies was the difference significant.^25^ Satisfaction and acceptability with the interventions was reported in four of the studies – all reported high satisfaction levels and acceptability of the interventions.^22–24,27^

**Table 5.** Main findings of the included studies.

Study, first author	HbA1c outcomes	Self-management outcomes	Satisfaction/acceptability
Agboola (2016)^22^	Significant decrease in HbA1c the IG by –0.43% (95% CI –0.75 to –0.12, *p* = 0.01), non-significant decrease in the CG. No significant difference in the change in HbA1c from baseline to follow up in the IG when compared to the CG. (0.07%; 95% CI –0.47 to 0.34, *p* = 0.75).	No statistically significant difference in overall **monthly step counts** between the IG and CG at follow up.	High ratings of usefulness, 94% would recommend it, and 72% wanted to continue the programme.
Arora (2014)^24^	Non-significant decreased in HbA1c by 1.05% in the IG compared with 0.60% in the CG (D0.45; 95% CI –0.27 to 1.17).	Non-statistically significant improvements in **medication adherence, knowledge, self-efficacy**, and **self-care activities (healthy eating, BG monitoring, foot care, exercise)** in the IG compared to CG.	Very high satisfaction, 100% would recommend it and 79% wanted to continue the programme.
Capozza (2015)^23^	Both groups average HbA1C decreased from baseline to follow up. No statistically significant difference between the IC and CG in terms of change in HbA1C at follow up (*p* > 0.05).	Not reported.	High satisfaction. 94% would recommend the programme to others.
Goodarzi (2012)^28^	The IG compared with CG improved significantly in HbA1C (*p* = 0.02).	Statistically significant improvements in **diet, physical activity, self-efficacy, practice** and **knowledge** in the IG. No significant improvement in **attitudes**.	Not reported.
Kim (2010)^25^	A significantly greater decrease in HbA1c from baseline to follow up was seen in the IG compared to the CG (*p* = 0.02).	Significantly higher BG **monitoring** during the study period in the IG group compared to the CG.	Not reported.
Lim (2016)^26^	A significant decrease in HbA1c from baseline to follow up was seen in the IG compared to the CG (*p* < 0.01).	Significantly greater decrease in **caloric intake** and increase in **exercise** episodes in the IG compared to CG.	Not reported.
Shetty (2011)^27^	There was no significant difference in the mean HbA1C values in both groups. The percentage of patients with HbA1c < 8% at one year increased significantly in the IG.	No significant improvement in **diet** or **physical activity**.	High acceptability based on the requested frequency of messages by participants.

BG: blood glucose; CG: control group; CI: confidence interval; HbA1c: haemoglobin A1c; IG: intervention group.

## Discussion

To our knowledge, this is the first systematic review to examine the use of SMS for delivery of diabetes self-management interventions specifically to those with poorly controlled diabetes. Seven RCTs met our criteria and were included in the review, with three of the studies reporting a significant decrease in HbA1c from baseline to follow-up in the intervention group compared with the control group.

Due to the small number and heterogeneity of the included studies, as well as the variable methodological quality of the trials, a meta-analysis of the data was not conducted and it is difficult to draw conclusions on the effectiveness of SMS interventions on glycaemic control in poorly controlled diabetes. Similarly, it is not possible to tease out the features/components of the SMS interventions that are associated with better outcomes. Unlike previous reviews reporting consistently positive impacts on glycaemic control for SMS interventions in patients with diabetes, when this review is limited to those with poorly controlled diabetes the evidence appears to be mixed. This review was also limited to the use of SMS messages that were automated rather than including those sent individually by a researcher or clinician. Use of individually sent (non-automated) SMS requires considerable cost and time, limiting its applicability for the wider population, and it could be argued that this is no different to individual clinician guidance provided via other mediums. As our review found mixed results, it could be further investigated whether individual clinician/researcher written feedback messages added to automated SMS interventions are needed to increase the effectiveness of the interventions for those with poorly controlled diabetes.

It has been reported that Internet and mobile-based interventions with a theoretical basis are more effective than those that have no theoretical basis.^29,30^ Two of the included studies reported a theoretical basis and neither of these studies found significant effects on their primary outcomes. Although the majority of studies did not explicitly state a theoretical basis, BCTs were utilised in all of the studies.

Interestingly, all four studies that reported no significant difference in the change in HbA1c between groups did report decreases in HbA1c in the intervention group over the study period. In addition, all four of these studies reported high acceptability and satisfaction with the interventions. This may indicate that this type of intervention is well received in the target population and provides some rationale for further development and investigation of SMS interventions in this group.

### Characteristics of effective interventions

The three interventions that found a significant decrease in HbA1c from baseline to follow-up were heterogeneous in their design. The first provided education and utilised SMS only, the second provided insulin adjustments based on patient-specific data gathered using a glucometer, and the final study provided medication and lifestyle guidance based on patient-specific data gathered using a glucometer and pedometer. A key similarity between two of the successful interventions was the use of devices to gather data to provide automated clinical guidance/feedback through SMS utilising BCTs, 2.4. Self-monitoring of outcome(s) of behaviour, and 2.7. Feedback on outcome(s) of behaviour. This monitoring functionality as well as individual feedback could be a key factor for success of mHealth interventions in this area.^31,32^ However, the inclusion of additional devices used for monitoring (e.g. glucometers and pedometers) adds further cost to the intervention which needs to be considered.

Dosages in the effective interventions varied from four messages per week through to eight messages per week. The self-management behaviours targeted also varied – one study only targeted taking medication whereas as the other two studies targeted taking medication, healthy eating, physical activity and monitoring. The effective interventions were all of short duration (three months or six months) with none providing long-term follow-up. Longer studies in this review did not show significant results, leading to questions about the sustainability of any significant findings.

With a lack of similarity between the three successful interventions, similarities between ineffective interventions was also explored. All four studies saw some degree of improvement in HbA1c in the intervention group. These studies were of longer duration, tailored, and had higher dose SMS. Interestingly, although the interventions in these studies were all well-received, all four studies had high rates of attrition which could be a contributing factor to the results. High attrition is common in mHealth studies,^33,34^ and ways to address this issue need to be considered.

### Limitations of review

This review has several limitations which must be considered. Key limitations include the small number of eligible studies and the methodological limitations of many of these studies. In addition only published full-text papers in English were included, resulting in potential for publication and language bias.

For this review, poorly controlled was defined as above the recommended target of 7% (53 mmol/mol). It is generally considered that a higher cut-off should be adopted for the definition of poorly controlled such as 8% (64 mmol/mol), therefore the findings in relation to ‘poor control’ must be interpreted with caution. However, if a higher cut-off had been utilised, only two studies would have met the criteria for the review, hence the benefit of the lower threshold.

A strength of this review is that it synthesises evidence from studies with RCT designs. Unfortunately, although all of the included studies were published from 2010 onwards (and four in the last three years), many of the articles failed to report key methodological features and detailed descriptions of the interventions. This is disappointing considering widely available guidance such as the Consolidated Standards of Reporting Trials (CONSORT) statement on how RCTs should be published.^35^ Therefore assessment of some types of bias in the included studies was unclear.

### Implications for future research

The findings from this review show potential for the utilisation of SMS in improving glycaemic control for those with poorly controlled diabetes, although more research is needed before recommendations can be made regarding adoption by healthcare services. Improving glycaemic control in those with poorly controlled diabetes is challenging but the benefits to success in this group are potentially great, both at an individual level and at a health system level. From this review it is unclear which characteristics and components of SMS interventions are more efficacious. This aligns with previous reviews highlighting that more work is needed to understand the successful components of this type of intervention.^36–38^ There is a need for better quality trials and more robust reporting on long-term follow-up.

Although this review excluded paediatric populations younger than 16 years (due to the unique characteristics of this group in managing diabetes) there was a lack of studies involving young adults (16–24 years). With both increasing prevalence of type 2 diabetes in young adults and the period of adolescence being a critical time for the formation of life-long habits around diabetes self-management in type 1 diabetes, there appears to be a need for more investigation of the use of mHealth in this group.

The content of the text messaging interventions is key to their success;^39,40^ mobile phones provide a platform, and SMS provides a delivery mechanism, for behaviour change interventions. However, this platform and mechanism are not a solution in themselves. This review was unable to demonstrate a relationship between positive outcomes and intervention content and functionality, and so more investigation needs to be made into what content and features are likely to be helpful. This investigation should include consideration of specific characteristics of people with poor control to ensure that interventions are personalised and tailored appropriately. Making them more relevant may also help to decrease attrition which is common in mHealth studies.

## Conclusions

The findings from the seven studies included in this review demonstrated that the evidence for improvements of SMS on glycaemic control in people with poorly controlled diabetes is mixed. Contrary to previous reviews reporting positive impacts on glycaemic control for SMS interventions in patients with diabetes, the evidence is less clear when review is limited to those with poorly controlled diabetes. The review is also limited by the small number of trials. Considering that diabetes management is one of the most investigated areas for the use of mHealth, this study highlights the lack of focus on those with poorly controlled diabetes, a group most in need of intervention.

## Supplementary Material

Supplementary material

## Supplemental Material

The online supplementary material is available at 10.1177/2055207617740315
